# Sequestration of 9-Hydroxystearic Acid in FAHFA (Fatty Acid Esters of Hydroxy Fatty Acids) as a Protective Mechanism for Colon Carcinoma Cells to Avoid Apoptotic Cell Death

**DOI:** 10.3390/cancers11040524

**Published:** 2019-04-12

**Authors:** Juan P. Rodríguez, Carlos Guijas, Alma M. Astudillo, Julio M. Rubio, María A. Balboa, Jesús Balsinde

**Affiliations:** 1 Instituto de Biología y Genética Molecular, Consejo Superior de Investigaciones Científicas (CSIC), Universidad de Valladolid, 47003 Valladolid, Spain; rodriguezcasco@me.com (J.P.R.); cguijas@ibgm.uva.es (C.G.); alma@ibgm.uva.es (A.M.A.); jrubio@ibgm.uva.es (J.M.R.); mbalboa@ibgm.uva.es (M.A.B.); 2 Laboratorio de Investigaciones Bioquímicas de la Facultad de Medicina (LIBIM), Instituto de Química Básica y Aplicada del Nordeste Argentino (IQUIBA-NEA), Universidad Nacional del Nordeste, Consejo Nacional de Investigaciones Científicas y Técnicas (UNNE-CONICET), Corrientes 3400, Argentina; 3 Centro de Investigación Biomédica en Red de Diabetes y Enfermedades Metabólicas Asociadas (CIBERDEM), 28029 Madrid, Spain

**Keywords:** colorectal cancer, hydroxystearic acid, fatty acid esters of hydroxy fatty acids, apoptosis

## Abstract

Hydroxy fatty acids are known to cause cell cycle arrest and apoptosis. The best studied of them, 9-hydroxystearic acid (9-HSA), induces apoptosis in cell lines by acting through mechanisms involving different targets. Using mass spectrometry-based lipidomic approaches, we show in this study that 9-HSA levels in human colorectal tumors are diminished when compared with normal adjacent tissue. Since this decrease could be compatible with an escape mechanism of tumors from 9-HSA-induced apoptosis, we investigated different features of the utilization of this hydroxyfatty acid in colon. We show that in colorectal tumors and related cell lines such as HT-29 and HCT-116, 9-HSA is the only hydroxyfatty acid constituent of branched fatty acid esters of hydroxyfatty acids (FAHFA), a novel family of lipids with anti-inflammatory properties. Importantly, FAHFA levels in tumors are elevated compared with normal tissue and, unlike 9-HSA, they do not induce apoptosis of colorectal cell lines over a wide range of concentrations. Further, the addition of 9-HSA to colon cancer cell lines augments the synthesis of different FAHFA before the cells commit to apoptosis, suggesting that FAHFA formation may function as a buffer system that sequesters the hydroxyacid into an inactive form, thereby restricting apoptosis.

## 1. Introduction

Colorectal cancer is a frequent cause of death due to late tumor presentation, rapid progression, and metachronous metastases [1]. Although hugely diverse in type and etiology, colon cancer cells often share as common characteristics a high rate of cell division and activated anti-apoptotic genes. In this regard, several studies have described mechanisms through which tumor cells escape apoptosis, some of which may involve lipid signaling [2,3,4]. 

Previous studies have shown that low micromolar concentrations of the hydroxy fatty acid 9-HSA exert cytostatic effects on highly proliferating cells such as human HT-29 colorectal cancer cells, causing cell arrest at the G_0_ phase of the cell cycle. At higher doses, 9-hydroxystearic acid (9-HSA) is a strong inducer of cell death by apoptosis. Increased 9-HSA levels may interfere with cell cycle kinetics by interacting with the cdc2 kinase complex and with p21, a potent cyclin-dependent kinase inhibitor controlled by the tumor suppressor protein p53 [5,6,7]. The effects of 9-HSA on cancer cell proliferation and differentiation have been attributed to inhibition of histone deacetylase-1 by a mechanism likely involving direct fatty acid/enzyme interaction. Such inhibition results in down-regulation of p53-dependent gene activation, driving cells to apoptosis [8,9,10]. In addition, 9-HSA-treated colon cancer cell lines have been found to be unable to re-enter the cell cycle and to proliferate in response to epidermal growth factor stimulation [9]. Collectively, these data raise the intriguing possibility that tumor cells may possess regulatory mechanisms to keep 9-HSA at low levels so as to avoid entering into apoptosis. 

**Figure 1:**
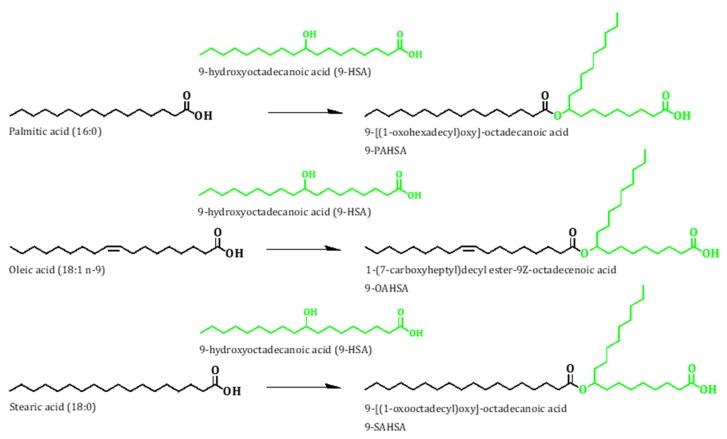
9-Hydroxystearic acid (9-HSA)-containing branched fatty acid esters of hydroxy fatty acids (FAHFA).

Recently, Yore et al. [11] described the existence of a novel family of lipids, called branched fatty acid esters of hydroxy fatty acids (FAHFA), and found that they were elevated in murine adipocytes overexpressing the GLUT-4 glucose transporter [12]. Structurally, FAHFA consists of a hydroxylated fatty acid, the hydroxyl moiety of which is esterified with palmitic acid (16:0), stearic acid (18:0), or oleic acid (18:1*n*−9) (Figure 1). Interestingly, the most abundant FAHFA in plasma and adipose tissue are those which contain 9-HSA, and 9-HSA-containing FAHFA possess anti-inflammatory properties and positively modulate insulin sensitivity and glucose tolerance in adipose tissue [11,12]. 

To the best of our knowledge, FAHFA have not been studied in tumor cells, and their possible effects on cell survival and proliferation have neither been documented. Due to the aforementioned pro-apoptotic properties of 9-HSA in colon cancer cells, the objective of this research was to investigate the biochemical and biological properties of this molecule in the development of colorectal cancer, using as a model both primary tumors and cell lines. Here, we identify FAHFA in human colon cancers and propose a novel mechanism by which colorectal cancer cells divert cytotoxic 9-HSA to non-toxic FAHFA as a strategy to escape from apoptosis.

## 2. Results and Discussion

**Figure 2:**
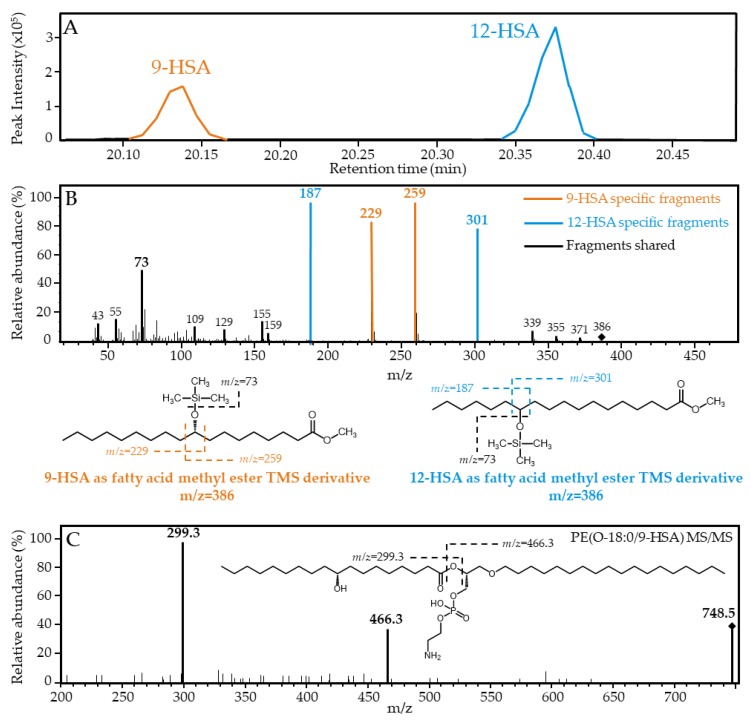
Characterization of free and esterified 9-HSA by mass spectrometry. (**A**) Gas chromatography (GC) separation of 9-HSA and 12-HSA methyl esters as trimethylsilyl (TMS) derivatives. (**B**) Electron impact fragmentation pattern of 9-HSA (orange) superimposed to the 12-HSA fragmentation pattern (blue). Due to structural similarity, both compounds share many fragments (black). However, the position of the hydroxyl group can be unambiguously established by the fragments *m/z* 229 and 259 for 9-HSA, and m/z 187 and 301 for 12-HSA. The other major fragment of m/z 73 is common to all TMS derivatives. (**C**) Collision-induced dissociation of the major phospholipid species PE(O-18:0/9-HSA), as analyzed by liquid chromatography/mass spectrometry (LC/MS). Fragments produced by the generation of the carboxylic anion of 9-HSA (m/z 299.3) and its neutral loss as a ketene (m/z 466.3) are observed, confirming that 9-HSA is esterified at the *sn-2* position of the glycerol backbone of the phospholipid molecule.

Growing evidence suggests that the monohydroxy fatty acid 9-HSA may inhibit cell proliferation by acting at different levels [5,6,7,8,9,10]. We began the current work by setting up conditions to identify and quantify 9-HSA in human colorectal cancer samples. Our initial assumption was that, in order to keep cell proliferation active, 9-HSA levels in cancer cells would be significantly lower than those of surrounding healthy tissue. Samples from colorectal cancer patients were obtained the day of scheduled surgery, and they were analyzed for hydroxylated fatty acid content by gas chromatography coupled to electron impact mass spectrometry (GC/MS). 9-HSA, as a trimethylsilyl (TMS) derivative, was found to resolve well in GC from 12-HSA, an isomer present at low levels in castor oil and other edible plant oils (Figure 2A). Since our own preliminary measurements confirmed that 12-HSA is not detectable in our samples (see below), we utilized this isomer as an internal standard for the quantification of 9-HSA. Figure 2B shows the merged electron impact fragmentation spectra of both 9-HSA and 12-HSA as TMS derivatives. Fragmentation of 9-HSA-TMS yielded the *m/z* 73 fragment that is common to all TMS derivatives, plus two diagnostic fragments of *m/z* 229 and 259, which represent the fragmentation of the molecule at both sides of the carbon carrying the hydroxyl group (Figure 2B, noted in orange). Similarly, 12-HSA-TMS yielded the *m/z* 73 fragment and two diagnostic fragments at *m/z* 187 and 301 (Figure 2B, noted in blue).

**Figure 3:**
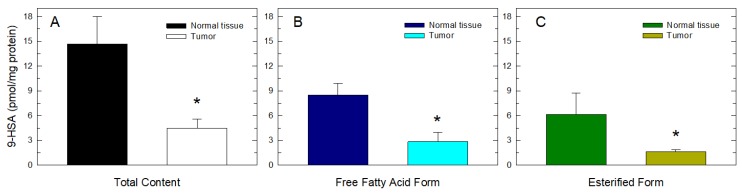
9-HSA levels are decreased in human colorectal cancer. Total 9-HSA (**A**) and 9-HSA present as free fatty acid (**B**) or esterified in phospholipids (**C**) in colon cancer samples vs. healthy adjacent tissue was determined by gas chromatography coupled to electron impact mass spectrometry (GC/MS) after lipid class separation and TMS derivatization. Data are expressed as means ± SEM (*n* = 11). * Significantly different (*p* < 0.05) from normal tissue.

Once this workflow was established, 9-HSA levels in colorectal cancer tumor samples were measured. Tumor samples were found to contain significantly lower amounts of 9-HSA than normal tissue samples (tissue sections adjacent to the tumor) (Figure 3). All tumor samples examined (*n* = 11) contained less 9-HSA than their normal adjacent counterparts, and this reduction affected both the free fatty acid as well as the phospholipid-esterified forms of the molecule (Figure 3). Importantly, no fragments other than those characteristic of 9-HSA (*m/z* 229 and 259) were identified in any of the samples analyzed, thus indicating that 9-HSA is the only hydroxylated fatty acid present at measurable quantities in our samples. Analysis of the distribution of esterified 9-HSA among cellular lipids revealed that the fatty acid was present only in phospholipids; 9-HSA was conspicuously absent of all neutral lipid classes, including mono- di- or tri-acylglycerol and cholesterol esters. 

**Figure 4:**
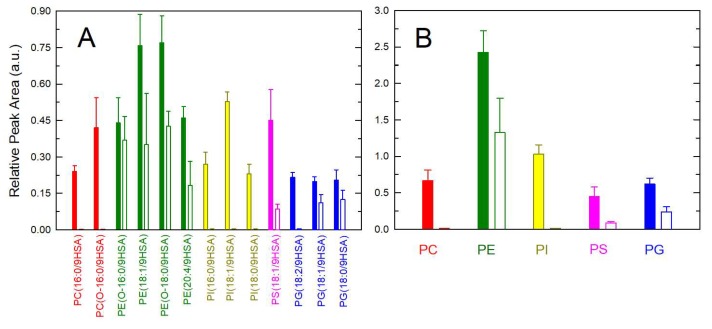
9-HSA-containing phospholipid species in human colorectal cancer. (**A**) The profile of 9-HSA-containing choline glycerophospholipids (PC, red), ethanolamine glycerophospholipids (PE, green), phosphatidylinositol (PI, yellow), phosphatidylserine (PS, pink) and phosphatidylglycerol (PG, blue) species in normal tissue (filled bars) or tumor (open bars) was determined by LC/MS/MS. (**B**) 9-HSA content in phospholipids as shown by class. Data are expressed as means ± SEM (*n* = 5).

In the next series of experiments, the distribution of 9-HSA among phospholipid molecular species was measured by liquid chromatography coupled to mass spectrometry (LC/MS). Identification of 9-HSA-containing species was carried out by determining the formation of a fragment of *m/z* 299, corresponding to the hydroxystearoyl fragment, in precursor-ion scan experiments. Structural identification of the glycerophospholipids was achieved by looking at the fragments and/or neutral losses obtained in product-ion scan experiments. Fatty chains within phospholipids are designated by their number of carbons:double bonds. A designation of O- before the first fatty chain indicates that the *sn-1* position is ether-linked [13]. Analysis of healthy tissue samples adjacent to the tumors revealed the presence of 13 glycerophospholipid species containing 9-HSA (Figure 4A). 

While 9-HSA was detected in all phospholipid classes examined, in relative terms, presence of the fatty acid in PE predominated. This is consistent with previous data in platelets showing that PE phospholipids are prone to contain oxidized fatty acids derived from arachidonic acid [14,15,16]. Consistent with the sharp decreases of 9-HSA already observed with GC/MS (Figure 3), tumor samples showed a general decrease in all phospholipid classes examined. The decrease was particularly pronounced in the case of PC and PI, which fell below detection levels in the tumor samples (Figure 4B). 

Although analyses by triple quadrupole mass spectrometry do not allow unambiguous assignation of a given fatty acid to either the *sn-1* or *sn-2* positions of the glycerol backbone, it is worth noting that three of the major 9-HSA-containing species detected are of the plasmanyl type (*sn-1* ether-linked) (Figure 4A). In these phospholipids, 9-HSA is necessarily present at the *sn-2* position, which raises the possibility that *sn-2* may constitute, in general terms, a preferred position for the fatty acid. In Figure 2C it was shown the MS/MS ion fragmentation of the major 9-HSA-containing species, PE(O-18:0/9-HSA). Note the fragment corresponding to 9-HSA as a carboxylate ion (*m/z* 299.3) and the ion resulting from the neutral loss as a ketene of the 9-HSA moiety (*m/z* 466.3). The existence of an alkyl ether bond at the *sn-1* position, which is not subjected to collision-induced dissociation at regular collision energies, confirms that 9-HSA is esterifying the *sn-2* position of the glycerol backbone of the phospholipid [17].

The presence of 9-HSA at the *sn*-2 position of some of the major phospholipid species detected could constitute an indication of the involvement of one or several phospholipase A_2_ enzymes in regulating the balance of 9-HSA between free fatty acid versus esterified forms [18,19]. Thus, the expression levels of the full set of phospholipase A_2_s (secreted: groups IIA to IIF, V and X; cytosolic: groups IVA to IVF; Ca^2+^-independent: groups VIA to VIF) [20,21] was analyzed by RT-PCR in normal and tumor samples. The oligonucleotide primers used for detection of the various phospholipase A_2_ genes have been described elsewhere [22]. Unfortunately, no clear and constant pattern of expression could be determined for any of the enzymes tested, so this approach was not pursued further.

**Figure 5:**
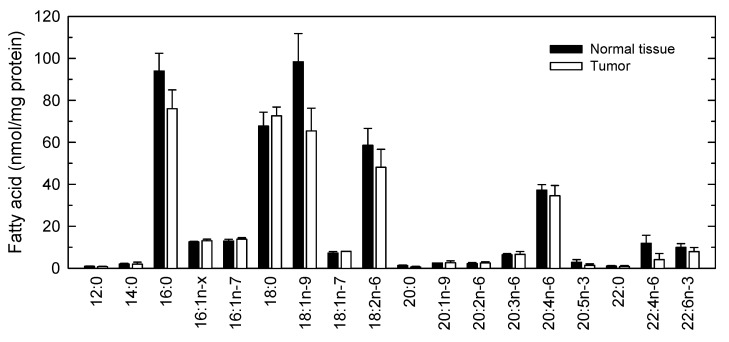
Phospholipid fatty acid composition of human colorectal tumors. The profile of major fatty acids in healthy normal tissue (black bars) or tumors (open bars) was determined by GC/MS after converting the fatty acid glyceryl esters into fatty acid methyl esters. 16:1*n*−x denotes a mix of the *n*−9 and *n*−10 isomers, which elute together. Data are expressed as means ± SEM (*n* = 17).

As our findings indicated that 9-HSA is present only in phospholipids, in the next series of experiments we investigated the phospholipid fatty acid composition of normal tissue and tumor samples by GC/MS (Figure 5). In both types of samples, palmitic acid (16:0), stearic acid (18:0), oleic acid (18:1*n*−9) and linoleic acid (18:2*n*−6) were the most abundant fatty acids, comprising nearly 75% of total fatty acid content. Interestingly, a marked reduction in the content of oleic acid (18:1*n*−9) could be appreciated in tumor samples compared to normal tissue. In nine out of 17 tumor samples examined, oleic acid levels were >50% lower than those found in their healthy tissue counterparts. Unfortunately, due to the large variations of oleic acid content between samples, the differences did not reach statistical significance. Smaller, non−statistically significant decreases in tumor samples were also detected for palmitic acid (16:0), linoleic acid (18:2*n*−6) and adrenic acid (22:4*n*−6). 

Interestingly, a recent Chinese study reported a significant reduction of oleic acid levels in colorectal cancer tumors [23]. This raises the intriguing possibility of whether low levels of oleic acid in colorectal tumors may bear a pathophysiological consequence and/or constitute a metabolic hallmark of the disease. Clearly, further investigation will be necessary to clarify these important aspects.

The sole presence of 9-HSA among hydroxy fatty acids in tumors raises another significant question as to what are the biochemical mechanisms that support the cellular levels of this fatty acid in cells, i.e., synthesis and clearance, and whether specific alteration of these routes could be envisioned as an effective manner to manipulate the levels of 9-HSA. Thus, the tendency of tumor samples to contain less oleic acid than adjacent normal tissue piqued our interest because of the simultaneous decrease of 9-HSA in tumors, which could be compatible with a possible precursor/product relationship between the two fatty acids. 

**Figure 6:**
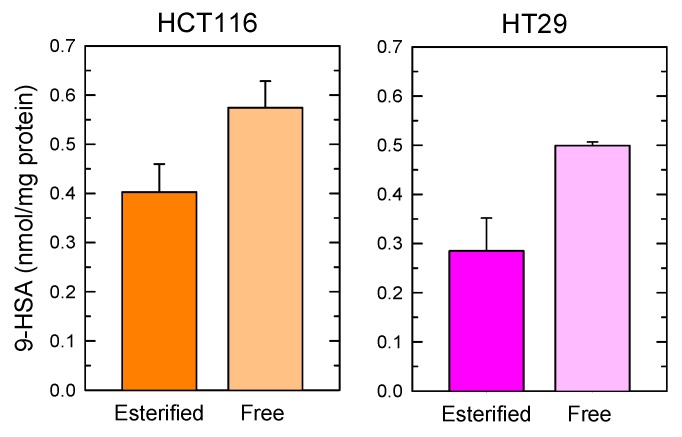
Determination of 9-HSA levels in colorectal cancer cell lines. The levels of both free fatty acid and esterified forms of 9-HSA in HCT116 cells (**left**) and HT29 (**right**) is shown. Data are expressed as means ± SEM (*n* = 3).

Some bacteria strains have been found to contain an enzyme, called oleate hydratase, capable of generating hydroxystearic acid via direct hydration of the double bond of oleic acid [24,25]. It is important to note, however, that the product of oleate hydration in bacteria is 10-HSA, not 9-HSA [24,25] and, as indicated above, 9-HSA is the only isomer found at measurable levels in tumors. To investigate whether 9-HSA is a metabolite of oleic acid in colorectal cancer, we incubated HT-29 or HCT-116 colorectal cancer cells with increasing amounts of [^2^H]oleic acid, and analyzed the possible appearance of 9-[^2^H]HSA by GC/MS and LC/MS at different times. HCT-116 and HT-29 cell lines are well established models for colon cancer with rapid proliferation rates, which offers greater versatility and more controlled conditions for assays than primary cultures of cells isolated from tumors, where cellular heterogeneity may reduce applicability. While both HT-29 and HCT-16 cells readily incorporated ^2^H-labeled oleic acid into phospholipids, no ^2^H-labeled HSA could be detected over a wide range of concentrations (up to 100 µM) and times (up to 40 h). Of note, both cells lines contained measurable levels of preexisting ‘endogenous’ (i.e., non-labeled) 9-HSA, both as free fatty acid and in esterified form (Figure 6). 

Next, we also examined the possibility that stearic acid, not oleic acid, was the biosynthetic precursor of 9-HSA. We labeled the cells with [^2^H]stearic acid and sought for 9-[^2^H]HSA by GC/MS and LC/MS. Again, our attempts were unsuccessful. Hence, we conclude that neither stearic nor oleic acid are biosynthetic precursors of 9-HSA in colon cancer cells. We also tested whether 9-HSA could be produced via non-enzymatic oxidation of oleic acid. Different oxidation methods were utilized, namely incubating aqueous dispersions of oleic acid (up to 100 µM) with the free radical generators 2,2′-azobis(2-methylpropionamidine) dihydrochloride, Fenton’s reagent (ferrous sulfate plus hydrogen peroxide), or just by exposing the oleic acid in air for at least 1 day. In neither case could 9-HSA be detected, suggesting that significant oxidation of oleic acid to 9-HSA is not attainable non-enzymatically. In addition, our preliminary attempts at establishing whether bacteria present in the gut microbiota make 9-HSA have been unsuccessful to this point.

All of the above negative results moved us to consider the possibility that, rather than being produced endogenously, 9-HSA arises from exogenous, dietary sources. If so, one would expect to measure significant levels of 9-HSA in serum. This happened to be the case, and we were able to estimate a 9-HSA concentration of 2.39 ± 0.36 μM in human serum, and 0.38 ± 0.05 µM in fetal calf serum (Gibco) (mean ± S.E.M., *n* = 3). These concentrations are sufficient to account for the levels of 9-HSA detected in tumors and adjacent normal tissue on the one hand, and in cultured HT-29 and HCT-116 cells on the other. Interestingly, 9-HSA in serum was found almost exclusively esterified in phospholipids, which suggests that the fatty acid may be more efficiently transported and delivered to cells and tissues in this manner. Consistent with our data suggesting a dietary origin for 9-HSA, studies by Wilson et al. [26] have demonstrated that the plasma concentration of hydroxy fatty acids increases in healthy women following their consumption, clearly indicating that they are effectively absorbed. 

**Figure 7:**
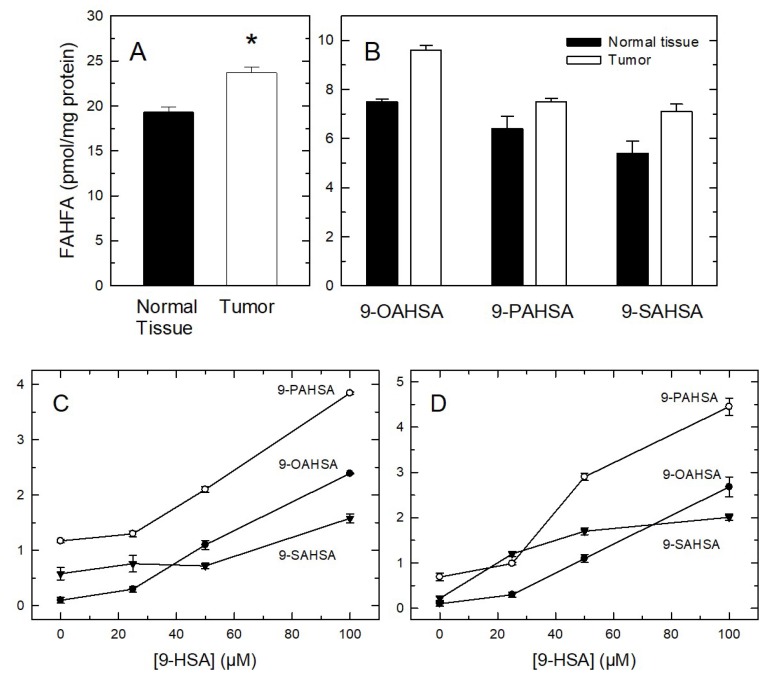
Determination of FAHFA levels in tumors and cell lines. (**A**) Total 9-HSA-containing FAHFA levels in normal tissue (black bar) or tumor (open bar) is indicated. (**B**) Identification of distinct FAHFA molecular species in normal tissue (black bars) and tumor (open bars). (**C**) FAHFA species present in HCT-116 cells increase when the cells are incubated with 9-HSA. (**D**) FAHFA species present in HT-29 cells increase when the cells are incubated with 9-HSA. Data are expressed as means ± SEM (*n* = 11 for tumors; *n* = 3 for cell lines). * Significantly different (*p* < 0.05) from normal tissue.

Recently, a new family of lipids consisting of FAHFA, with important pathophysiological implications, has been described [11,12]. These compounds are of biomedical interest because of their beneficial actions on a number of inflammatory and metabolic diseases, and attracted our attention because they contain 9-HSA as a major constituent [11,12,27]. FAHFA have been shown to be produced in vivo from preexisting hydroxy fatty acids via a putative acyl-CoA acyltransferase-like reaction [27,28]. Although the biochemical origin of the constituent hydroxy fatty acid has not been defined, evidence has been provided that the synthesis is stereospecific, as the *R*-isomer appears to be preferred over the *S*-isomer. This implies again the involvement of enzymes in FAHFA synthesis [29]. We were able to detect three 9-HSA-containing FAHFA in our colorectal tumor samples and adjacent normal tissue, namely those esterifying oleic, palmitic, and stearic acids: 9-OAHSA (oleic acid ester of 9-hydroxystearic acid), 9-PAHSA (palmitic acid ester of 9-hydroxystearic acid) and 9-SAHSA (stearic acid ester of 9-hydroxystearic acid), respectively (Figure 7A,B). Strikingly, the amount of FAHFA in tumors was found to be significantly elevated when compared to normal tissue (Figure 7A,B).

**Figure 8:**
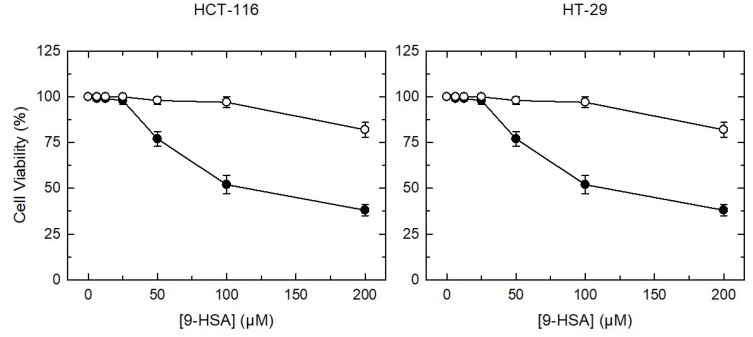
Concentration-dependence of the effect of 9-HSA on cell viability. HT-116 cells (**left**) or HT-29 cells (**right**) were incubated with the indicated concentrations of 9-HSA (black circles) or oleic acid (open circles), as a negative control for 24 h. Cell viability was measured with the CellTiter 96^®^ AQ_ueous_ One Solution Cell Proliferation Assay kit. Data are expressed as means ± SEM (*n* = 3).

The same three FAHFA species were also found in HCT-116 (Figure 7C) and HT-29 cells (Figure 7D), albeit in the cell lines the major species was 9-PAHSA, not 9-OAHSA as in tumors. Interestingly, when both cell lines were incubated with increasing amounts of 9-HSA, the cellular levels of the three FAHFA increased accordingly (Figure 7C,D). The latter is an important finding because it clearly indicates that FAHFA formation may constitute a cellular strategy to dampen the cellular level of 9-HSA in the form of free fatty acid, which is toxic to cells. To assess this possibility, we characterized 9-HSA-induced cell death in colon cancer cells. Figure 8 shows that incubation of either HCT-116 or HT-29 cells with 9-HSA concentrations ≥50 µM, resulted in marked losses of cell viability, as measured by the CellTiter 96^®^ AQ_ueous_ One Solution Cell Proliferation Assay kit. Cells exposed to the same concentrations of oleic acid as a control, experienced no such viability losses. This range of concentrations at which 9-HSA induces cell death is consistent with previous reports utilizing various cell types [6,7,8,9,10].

**Figure 9:**
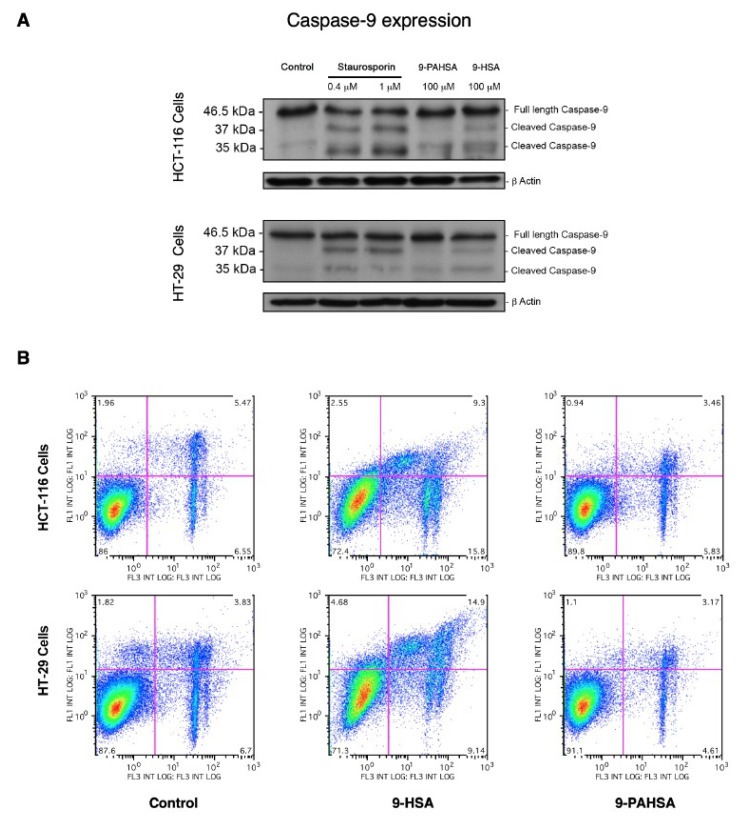
Analysis of apoptotic markers in 9-PAHSA (palmitic acid ester of 9-hydroxystearic acid) and 9-HSA-treated HCT-116 and HT-29 cells. (**A**) Untreated cells or cells treated with either 100 µM 9-HSA or 100 µM 9-PAHSA for 24 h, were harvested and protein was separated by SDS-polyacrylamide gel electrophoresis, and analyzed by Western blotting. Cleaved fragments of caspase 9 are indicated. Blots were re-probed for β-actin to confirm equal loading of the samples. (**B**) Flow cytometry-based apoptosis detection. Apoptotic changes in plasma membrane were detected by simultaneous staining with annexin V-fluorescein isothiocyanate (FITC) (FL1) and propidium iodide (PI; FL3). HCT-116 and HT-29 cells were untreated (Control) or treated with either 100 µM 9-HSA or 100 µM 9-PAHSA for 24 h. Cells were collected, and their green and red fluorescence was measured by flow cytometry. Live cells are both annexin V and propidium iodide (PI) negative. At early stage of apoptosis, cells bind annexin V while still excluding PI (upper left quadrant). At late stage of apoptosis, they bind annexin V-FITC and stain brightly with PI (upper right quadrant). Primary necrotic and some very late apoptotic cells stain with PI only (lower right quadrant). These data are representative of three independent experiments.

The effect of 9-HSA on caspase-9 fragmentation was analyzed by immunoblot, and the results are shown in Figure 9A. Staurorporine was used in these experiments as a positive control. The results clearly showed that 9-HSA induced caspase-9 fragmentation. In addition, we noted that treating the cells with 9-HSA also resulted in the caspase-3-dependent cleavage of poly(ADP-ribose) polymerase 1 substrate, which was observed as early as 4 h after exposure of the cells to the fatty acid, in agreement with previous estimates in other cells types [30,31]. Importantly, 9-PAHSA, used at the same concentration as 9-HSA and under identical experimental conditions, failed to show any effect. Similar results were obtained when flow cytometry-based detection assays of loss of membrane asymmetry were carried out. HCT-116 and HT-29 cells were either untreated or treated with 9-HSA or 9-PAHSA (Figure 9B). Untreated cells retained the same appearance as live cells (without annexin-V or propidium iodide staining), and the same was true for the 9-PAHSA treated-cells. In both cases, the upper quadrants of the cytometry analysis (left and right) for both cell lines, showed less than 5% of cell death due to early or late apoptosis. In contrast, 9-HSA treated-cells showed apoptotic cell death close to 12% (HCT-116) or 20% (HT-29). Collectively, these data support the concept that incorporation of 9-HSA into FAHFA abrogates the ability of the hydroxy fatty acid to induce apoptotic cell death. 

## 3. Materials and Methods

### 3.1. Reagents

Cell culture medium was from Molecular Probes-Invitrogen (Carlsbad, CA, USA). Organic solvents (Optima^®^ LC/MS grade) were from Fisher Scientific (Madrid, Spain). Lipid standards were purchased from Avanti (Alabaster, AL, USA) or Cayman (Ann Arbor, MI, USA). Deuterated fatty acids (stearic-d35 acid and oleic-d34 acid) were from Sigma-Aldrich. SilicaGel thin-layer chromatography plates were from Macherey-Nagel (Düren, Germany). 9-HSA and 12-HSA were synthesized and provided by Dr. Alfonso Pérez (Department of Organic Chemistry, University of Valladolid, (Valladolid, Spain). All other reagents were from Sigma-Aldrich (Madrid, Spain).

### 3.2. Patients and Sampling Procedures

Samples of Stage I or Stage IIA colorectal cancer, which otherwise would have been discarded, were obtained at the time of surgery from patients undergoing colon resection at the Clinical Hospital of the University of Valladolid School of Medicine. Adjacent grossly normal-appearing tissue specimens to be used as controls were collected from the accompanying normal mucosa, distant at 10–15 cm from the carcinoma. The study was approved by the Research Ethics Committee of the Clinical Hospital of the University of Valladolid School of Medicine (date of approval, February 23, 2013), in accordance with the guidelines established by the Spanish Ministry of Health and the European Union, and in conformity with the World Medical Association Declaration of Helsinki. Written informed consent was obtained from each donor. The researchers received the samples in an anonymous manner.

### 3.3. Cell Lines

The human colon cancer cell lines HCT-116 and HT-29 were provided by Dr. Ana B. Herrero (Instituto de Biología Molecular y Celular del Cáncer, Consejo Superior de Investigaciones Científicas, Salamanca, Spain). They were maintained in Dulbecco’s Modified Eagle medium supplemented with 10% (v/v) fetal bovine serum, 2 mM glutamine, penicillin (100 units/mL), and streptomycin 100 μg/mL. The cells were incubated at 37 °C in a humidified atmosphere of 5% CO_2_. Cell viability was measured using the CellTiter 96^®^ AQ_ueous_ One Solution Cell Proliferation Assay kit (Promega, Madison, WI, USA), following the manufacturer’s instructions.

### 3.4. Detection of Apoptosis

The effect of 9-HSA or 9-PAHSA on apoptosis was evaluated over a wide range of concentrations (1–400 μM). Based on preliminary time-course data, the exposure time was set to 24 h, and apoptosis was analyzed by labeling with the annexin V-fluorescein isothiocyanate (FITC) apoptosis detection kit (BD Bioscience, San Jose, CA, USA), which recognizes phosphatidylserine exposure on the outer leaflet of the plasma membrane. After washing the cells, cell fluorescence was quantified by flow cytometry in FL1 (Gallios; Beckman Coulter, Barcelona, Spain). Data were analyzed with the FlowJo software version 8.7. Propidium iodide (PI, Sigma-Aldrich, Madrid, Spain) uptake was analyzed by incubating cells with 50 μg/mL PI in PBS in the dark for 5 min. Fluorescence was quantified by flow cytometry in FL3. Data were analyzed with FlowJo version 8.7. In parallel to these measurements, the cells, treated with 9-HSA or 9-PAHSA for the time indicated, were analyzed by immunoblot for the intact and cleaved forms of caspase-9. 

### 3.5. Immunoblot Analyses 

After the different treatments, the cells were lysed in ice-cold buffer containing 20 mM Tris-HCl (pH 7.4), 150 mM NaCl, 0.5% Triton X-100, 100 mM Na_3_VO_4_, 1 mM phenyl methyl sulfonyl fluoride, and protease inhibitor cocktail (Sigma). Total protein (50 μg) was resolved on 10–12% SDS-PAGE gels and transferred to a nitrocellulose membrane. After transfer, nonspecific binding sites were blocked with 5% defatted dry milk in PBS containing 0.1% Tween-20 at room temperature for 1 h. The membranes were then probed with the corresponding antibodies, followed by HRP-conjugated secondary antibodies in blocking solution. β-Actin was used as a load control. The immunoblots were visualized using enhanced luminescence.

### 3.6. Real-Time PCR

Total RNA was extracted using Trizol reagent (Ambion-Thermo Fisher, Waltham, MA, USA). The cDNA templates were synthesized using Verso cDNA synthesis kit (Thermo Fisher Scientific, Waltham, MA, USA), following the manufacturer’s instructions. Quantitative PCR (qPCR) was carried out with a 7500 Real Time PCR System (Applied Biosystems, Carlsbad, CA, USA), using Brilliant III Ultra-Fast SYBR Green qPCR Master Mix (Agilent Technologies, Santa Clara, CA, USA), as previously described [32].

### 3.7. Gas Chromatography/Mass Spectrometry (GC/MS) Analyses

Tumor samples were sliced, weighed and homogenized mechanically with a polytron tissue homogenizer prior to total lipid extraction according to Folch et al. [33]. Cultured cell samples (approx. 10^7^ cells) were washed with PBS, lysed with water, and sonicated in a tip homogenizer twice for 15 s, prior to total lipid extraction according to Bligh and Dyer [34]. The following internal standards were added before lipid extraction: 10 nmol of 1,2-diheptadecanoyl-*sn*-glycero-3-phosphocholine, 10 nmol of 1,2,3-triheptadecanoyl-*sn*-glycerol and 30 nmol of cholesteryl tridecanoate. Lipid classes were separated by thin-layer chromatography, using *n*-hexane/diethyl ether/acetic acid (70:30:1, v/v/v) as the mobile phase [35]. Glycerolipids and glycerophospholipids were transmethylated with 500 μL of 0.5 M KOH in methanol for 30 min at 37 °C, and 500 μL of 0.5 M HCl was added to neutralize. For the transmethylation of cholesterol esters, the samples were resuspended in 400 μL methyl propionate, and 600 μL 0.84 M KOH in methanol for 1 h at 37 °C. Afterwards, 50 and 1 mL of acetic acid and water, respectively, were added to neutralize [36,37,38].

Hydroxylated fatty acids need an extra derivatization step to introduce a trimethylsilyl group that reduces lability of the hydroxyl groups and makes the molecule volatile for GC/MS analysis. Fatty acid methyl esters obtained as described above were used as starting material for trimethysilylation. A total of 150 μL of Tri-Sil HTP (HDMS:TMCS:pyridine) Reagent (Thermo Scientific) was added to the fatty acid methyl ester extract. Samples were vortexed for 30 s and the derivatization reaction was carried out for 10 min at room temperature under continuous stirring. The solvent was evaporated and 500 μL water and 800 µL *n*-hexane was added. After vigorous vortexing for 30 s, the upper organic layer was saved.

Analysis of fatty acid methyl esters, either derivatized with TMS or not, was carried out using an Agilent 7890A gas chromatograph coupled to an Agilent 5975C mass-selective detector operated in electron impact mode (EI, 70 eV) equipped with an Agilent 7693 autosampler and an Agilent DB23 column (60 m length × 250 µm internal diameter × 0.15 µm film thickness) under the conditions described previously [38,39]. Data analysis was carried out with the Agilent G1701EA MSD Productivity Chemstation software, revision E.02.00 (Santa Clara, CA, USA).

### 3.8. Liquid Chromatography/Mass Spectrometry (LC/MS) Analyses

Lipids were extracted according to Bligh and Dyer [34], and the following internal standards were added: 20 pmol each of 1,2-dimyristoyl-*sn*-glycero-3-phosphoglycerol, 1,2-dilauroyl-*sn*-glycero-3-phosphoethanolamine, 1,2-dimyristoyl-*sn*-glycero-3-phosphoethanolamine, 1,2-di-heptadecanoyl-*sn*-glycero-3-phosphoethanolamine, 1,2-dimyristoyl-*sn*-glycero-3-phosphoserine, 1,2-dimyristoyl-*sn*-glycero-3-phosphate, 1,2-dipentadecanoyl-*sn*-glycero-3-phosphocholine, 1,2-di-heptadecanoyl-*sn*-glycero-3-phosphocholine and 1,2-dinonadecanoyl-*sn*-glycero-3-phosphocholine. The samples were redissolved in 50 μL of hexanes/2-propanol/water (42:56:2, v/v/v), and 40 μL was injected into an Agilent 1260 Infinity high-performance liquid chromatograph equipped with an Agilent G1311C quaternary pump and an Agilent G1329B autosampler. The column was a FORTIS HILIC (150 × 3 mm, 3 μm particle size) (Fortis Technologies, Geston, UK), protected with a Supelguard LC-Si (20 × 2.1 mm) cartridge (Sigma-Aldrich). The mobile phase consisted of a gradient of solvent A (hexanes/2-propanol, 30:40, v/v) and solvent B (hexanes/2-propanol/20 mM ammonium acetate in water, 30:40:7, v/v/v). The gradient started at 75% A from 0 to 5 min, then decreased from 75% A to 40% A at 15 min, from 40% A to 5% A at 20 min, holding at 5% until 40 min, and increasing to 75% at 41 min. The column was then re-equilibrated by holding at 75% A for an additional 14 min before the next sample injection. The flow rate through the column was fixed at 400 μL/min, and this flow entered into the electrospray ionization interface of an AB/Sciex QTRAP 4500 hybrid triple quadrupole mas spectrometer operated in negative ion mode. Source parameters were as follows: ion spray voltage, −4500 V; curtain gas, 30 psi; nebulizer gas, 50 psi; desolvation gas, 60 psi; temperature, 425 °C. Phospholipid species were detected as [M−H]^−^ ions except for choline phospholipids, which were detected as [M+CH_3_COO^−^]^−^ adducts, and were identified by comparison with previously published data [40,41,42,43,44,45,46].

FAHFA analysis by LC/MS was carried out as described by Zhang et al. [47]. Briefly, total lipid extracts were redissolved in 500 μL chloroform and loaded into solid-phase extraction silica columns (Supelco Discovery DSC-Si, Sigma-Aldrich) that had been previously conditioned by passing 15 mL *n*-hexane through them. Neutral lipids were eluted with 5 mL *n*-hexane/ethyl acetate (95:5, by vol.). FAHFA were eluted with 4 mL ethyl acetate and evaporated under a nitrogen stream. Quantitative isolation of FAHFAs by this method was confirmed by using an authentic 9-PAHSA standard. The FAHFA fraction was redissolved in 50 µL methanol, and 40 µL was injected into an Agilent 1260 Infinity high-performance liquid chromatograph equipped with an Agilent G1311C quaternary pump and an Agilent G1329B autosampler. The column was a reversed-phase C18 column (250 × 2 mm, 5 µm particle size, Knauer, Berlin, Germany). Total flow through the column was 400 µL/min, with 93:7 MeOH:H_2_O with 5 mM ammonium acetate and 0.01% ammonium hydroxide as solvent. Total run time was 60 min. The LC system was coupled to an AB/Sciex API 4500 Q-TRAP hybrid triple quadrupole mas spectrometer operated in negative ion mode. Source parameters were as follows: ion spray voltage, −4500 V; curtain gas, 30 psi; nebulizer gas, 50 psi; desolvation gas, 60 psi; temperature, 500 °C. For the quantitative measurement of 9-HSA-containing FAHFA species (especially those containing palmitic, palmitoleic, stearic, oleic and linoleic acids), the instrument was set to multiple reaction monitoring mode, selecting three transitions for each FAHFA: parent ion → 299.3 (9-HSA), parent ion → 281.3 (9-HSA as water loss) and parent ion → *m/z* of esterified fatty acid as carboxylate ion. Only peaks with a signal-to-noise ratio higher than five were quantified.

### 3.9. Statistical Analysis

Data are expressed as means ± SE of the number of independent determinations indicated. Student’s *t*-test was applied for statistical analysis of the data, with *p* < 0.05 taken as statistically significant.

## 4. Conclusions

We report in this study that human colorectal tumors contain significantly lower levels of 9-HSA than adjacent normal tissue, suggesting that highly proliferating cells such as those of tumors possess mechanisms to keep 9-HSA at low levels and, in this manner, inactivate an endogenous brake to uncontrolled cell division. Our results add to this concept by demonstrating that colorectal cancer lines make significant amounts of various FAHFA when supplied with 9-HSA. Further, we made the key observation that, while 9-HSA is pro-apoptotic, 9-HSA-containing FAHFA is not. Thus, in addition to its well described roles as anti-diabetic and anti-inflammatory lipids, FAHFA may be produced as an anti-apoptotic strategy to ameliorate the pro-apoptotic effects of hydroxy fatty acids accumulating in cells. 

In accord with all the above, we demonstrate as well that FAHFA content in human tumor samples is significantly elevated when compared with adjacent normal tissue, clearly suggesting a pathophysiological role, such as preventing the pro-apoptotic effects of 9-HSA. Thus, FAHFA synthesis may occur at proliferative tumor stages where there is a predominance of mitosis over apoptosis and, in opposition, cells may activate FAHFA hydrolysis at times of declining cell death, thus regulating the cell cycle via lipid signaling. The finding that FAHFA molecules are found in tumors shows that this mechanism may work in vivo and, at the same time, provides an interesting starting point from which to consider these lipids as pharmacological targets, focusing on the specific hydrolysis of FAHFA that provides apoptotic hydroxyacids in situ.
